# Effects of intestinal flora on polycystic ovary syndrome

**DOI:** 10.3389/fendo.2023.1151723

**Published:** 2023-03-09

**Authors:** Jiayue Liu, Ying Liu, Xiaoliang Li

**Affiliations:** ^1^ Key Laboratory of Tropical Translational Medicine of Ministry of Education, Hainan Provincial Key Laboratory for Research and Development of Tropical Herbs, Haikou Key Laboratory of Li Nationality Medicine, School of Pharmacy, Hainan Medical University, Haikou, China; ^2^ Key Laboratory of Tropical Cardiovascular Diseases Research of Hainan Province, Cardiovascular Diseases Institute of the First Affiliated Hospital, Hainan Medical University, Haikou, China; ^3^ College of Pharmacy, Heilongjiang Provincial Key Laboratory of New Drug Development and Pharmacotoxicological Evaluation, Jiamusi University, Jiamusi, Heilongjiang, China

**Keywords:** intestinal flora, polycystic ovary syndrome, hyperandrogenemia, insulin resistance, chronic inflammation, obesity

## Abstract

Polycystic ovary syndrome (PCOS) is a common endocrine disorder in women of reproductive age. Its clinical characteristics are mainly oligo-ovulation or anovulation, hyperandrogenemia (HA) and insulin resistance (IR). PCOS is considered to be one of the main causes of infertility in women of childbearing age, and its pathogenesis is still unclear. Intestinal flora, known as the “second genome” of human beings, is closely related to metabolic diseases, immune diseases and infectious diseases. At the same time, mounting evidence suggests that intestinal flora can regulate insulin synthesis and secretion, affect androgen metabolism and follicular development, and is involved in the occurrence of chronic inflammation and obesity. The imbalance of intestinal flora is caused by the abnormal interaction between intestinal flora and host cells caused by the change of intestinal microbial diversity, which is related to the occurrence and development of PCOS. The adjustment of intestinal flora may be a potential direction for the treatment of PCOS.

## 1 Introduction

PCOS is a common endocrine disorder in women. The prevalence rate of PCOS among women of childbearing age is 5% ~ 10% globally, and it is increasing year by year (1–3). PCOS are mainly manifested by irregular menstruation or infertility, hirsutism, acne, obesity, HA, IR, enlargement and polycystic changes of the ovaries (4–6). Currently, the Rotterdam criteria are commonly used for the diagnosis of PCOS in clinical practice. According to this criteria, the serum androgen level of patients with PCOS is remarkable increased, ovulation is significantly decreased, and polycystic ovary appear. If two of the above criteria are met, they can be classified as PCOS (7). Besides, PCOS is a high risk factor for diabetes, metabolic syndrome, endometrial cancer, cardiovascular and cerebrovascular diseases and other diseases, which seriously affects the health of women (8–10). Up to now, it is generally believed that PCOS is a disease caused by multiple factors. Its etiology and pathogenesis usually involve genetics, inflammatory factors, intestinal flora, endocrine hormones and IR (11–13). In recent years, the study of intestinal flora in patients with PCOS has attracted widespread attention, and it has been found that intestinal flora plays a key role in the occurrence and development of PCOS (14, 15).

The human gut is home to trillions of microorganisms, including bacteria, archaea, fungi, protists and viruses, of which bacteria are the main “residents” (16, 17). These bacteria contain 800 species and more than 7,000 strains, about 1014, with a total mass of 1 ~ 2 kg, which is known as the second genome of human (18). The microorganisms living in the gastrointestinal system of the host mainly rely on the digestion of the food residues in the host body to provide energy for themselves, and these microorganisms together with their living environment constitute the intestinal microecosystem (19–21). When the number of harmful bacteria in the gut increases, it will cause physical discomfort, and even cause serious inflammatory and immune responses (22). When the body’s metabolic dysfunction, it is easy to lead to the loss of bacteria with protective effect in the intestinal tract, which will cause changes in the composition of microorganisms in the intestine, and finally, the intestinal barrier is destroyed. Recent studies have shown that changes in intestinal flora are common in patients with PCOS. Moreover, the imbalance of intestinal microecology is related to the occurrence and progression of PCOS, and intestinal flora is involved in the pathological links of PCOS, such as HA, IR, chronic inflammation, obesity, etc (23–26). Based on the etiology and pathogenesis of PCOS in recent years, this article reviewed the research progress of the relationship between intestinal flora and PCOS.

## 2 Relationship between intestinal microecological disorders and HA in PCOS

### 2.1 HA and PCOS

The abnormality of sex hormones is an important feature of PCOS, in which the clinical or biochemical manifestations of HA are the main, which is also belong to the core pathological manifestations of PCOS. Hormonal abnormalities in PCOS patients are mainly manifested by elevated testosterone levels (27). Some scholars pointed out that the possibility of PCOS caused by excessive androgen secretion is 82% through the study of more than 1200 women with high androgen levels (3). The synthesis of androgens in women is closely related to the hypothalamus-pituitary-ovarian axis. The hypothalamus secretes gonadotropin-releasing hormone (GnRH), which leads to the release of luteinizing hormone (LH) and follicle-stimulating hormone (FSH). LH acts on theca cells to synthesize androgen, while FSH acts on granulosa cells to convert androgen into estrogen (28). The mechanism of androgen increase is that LH stimulates the transformation of cholesterol in the follicular membrane cells into pregnenolone through cytochrome P450 side chain lyase, which in turn synthesizes androstenedione, and then finally convert into testosterone *via* 17P-hydroxysteroid dehydrogenase (29). The androgen in thecal cells diffuses to granulosa cells, FSH stimulates aromatase activity located in granulosa cells, and eventually converts it into estradiol. The increase of LH level can not only increase the androgen from the ovary, but also reduce the FSH level through the negative feedback effect of estrogen, resulting in leads to the decrease of aromatase activity and the reduction of androgen to estrogen conversion. However, the exposure of immature oocytes to high androgen levels will cause follicular growth arrest or even atresia, which is the occurrence of ovulation disorder (30). Similarly, low level of FSH and insufficient conversion of estradiol also lead to the occurrence of this process (31). In addition, in the occurrence and development of PCOS, HA and IR are closely connected and promote each other, eventually forming a vicious cycle (32).

### 2.2 Relationship between intestinal flora and HA in PCOS

**Figure 1:**
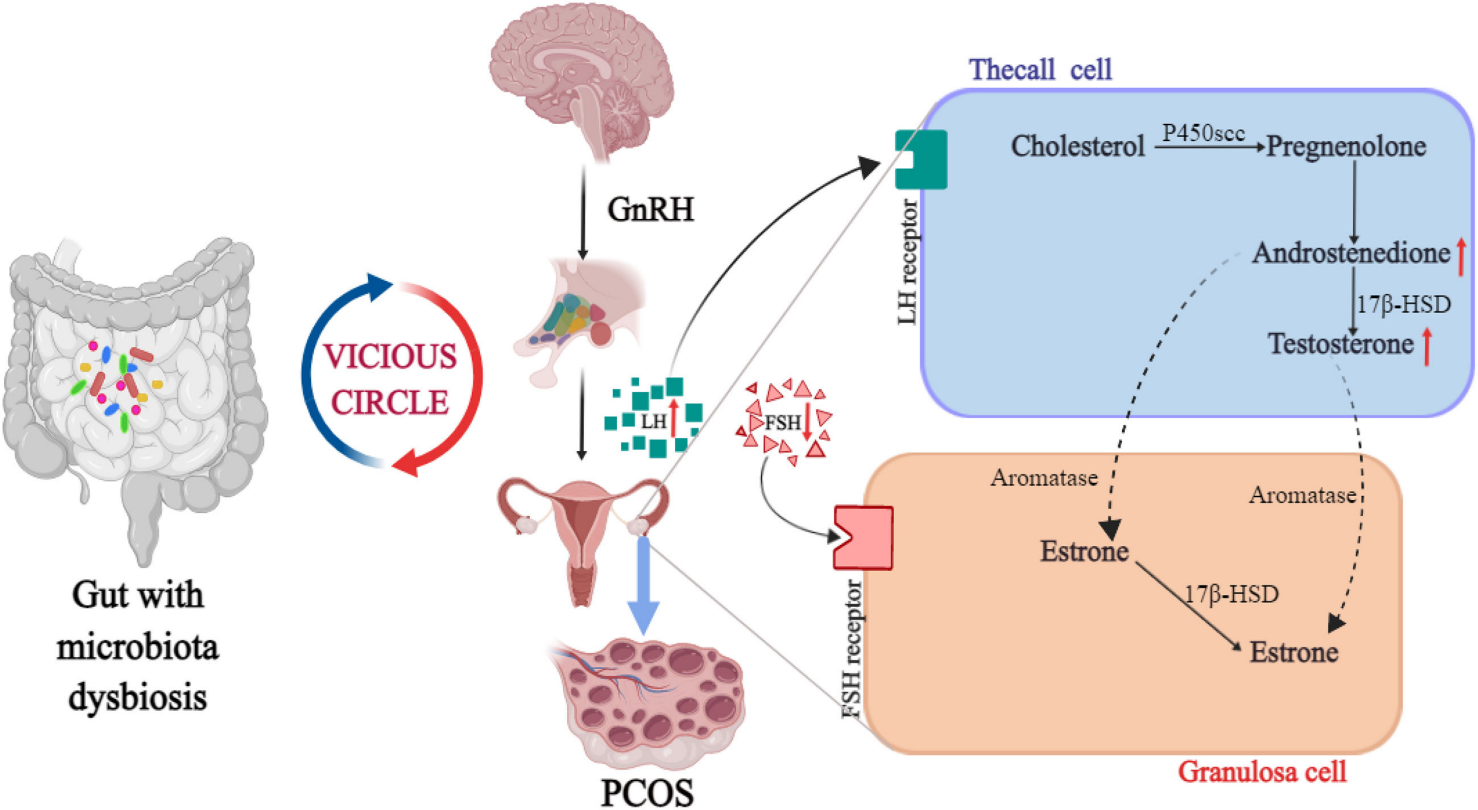
The intrinsic relationship between HA, intestinal flora and PCOS.

Gut microbiota can change in response to changes in hormone levels, which in turn affect sex hormone levels in the body. The research on the relationship between sex hormones and intestinal flora mostly focused on the serum testosterone. It was found that serum testosterone and hirsutism were negatively correlated with α diversity of flora (33, 34), while the level of free testosterone was correlated to the ratio of *Firmicutes/Bacteroidetes* (35). In the study of PCOS mouse model induced by letrozole, HA was found to reduce the species and the number of bacteria in the large intestine of mice. The main results showed that the number of *Bacteroides* decreased, the number of *Firmicutes* increased, body mass, fat mass and blood glucose level increased compared with the control group, indicating that HA can significantly change the intestinal flora (36). In a non-obese diabetic mouse model, it was found that the microbial changes in the intestinal flora could affect the sex hormone level of mice. Transplanting the intestinal flora of mature male mice into the body of immature female mice increased testosterone levels in the female mice (37). In another study, the serum testosterone level of mice fed with lactobacillus was remarkably higher than that of untreated mice. At the same time, the testis of mice was significantly enlarged, the number of spermatogenesis and testicular interstitial cells increased. This study indicated that the change of intestinal flora can regulate the serum testosterone level, affect the change of metabonomics, and promote the occurrence of islet inflammation (38). The above studies showed that HA may interact with intestinal flora in the pathophysiological process of PCOS. The intestinal flora not only affects the level of androgen, but is regulated by androgen in turn, as shown in **Figure 1**.

## 3 Relationship between intestinal microecological disorders and IR in PCOS

### 3.1 IR and PCOS

IR is one of the pathophysiological mechanisms leading to PCOS. 50%-70% of PCOS patients are accompanied by IR and compensatory hyperinsulinemia (32, 39, 40). Insulin is a polypeptide secreted by pancreatic β cells, consisting of 51 amino acids. Its physiological function is to regulate the metabolism and gene expression of the body by activating PI3K/PKB and MAPK/Ras signal pathways after binding to insulin receptor (41). When the insulin receptor and corresponding signal pathway are interfered, the sensitivity of peripheral tissue to insulin decreases, which leads to the obstruction of glucose utilization in peripheral tissues. In order to regulate the body’s blood sugar level, the body will secrete insulin compensatively, resulting in high blood insulin level, that is, IR. Glucose metabolism can directly provide energy for follicular growth. Therefore, abnormal glucose metabolism caused by IR will affect follicular growth and ovulation in PCOS (40), which inevitably affects the normal physiological function of ovary. The increase of fasting insulin level can trigger the insulin receptor of the pituitary gland and stimulate the secretion of LH by the pituitary. In addition, IR can also enhance the effect of cytochrome P450C17a enzyme in theca cells through insulin-like growth factor to improve the level of androgen (42). The elevated androgen can cause symptoms such as hirsutism, acne and alopecia in PCOS patients. More seriously, the increase of local androgen in the ovary can cause premature follicular atresia and the formation of dominant follicles, resulting in ovulation dysfunction. Meanwhile, the increase of androgen level further promotes the development of IR, thus forming a vicious cycle and aggravating the process of PCOS.

### 3.2 Relationship between intestinal flora and IR in PCOS

**Figure 2:**
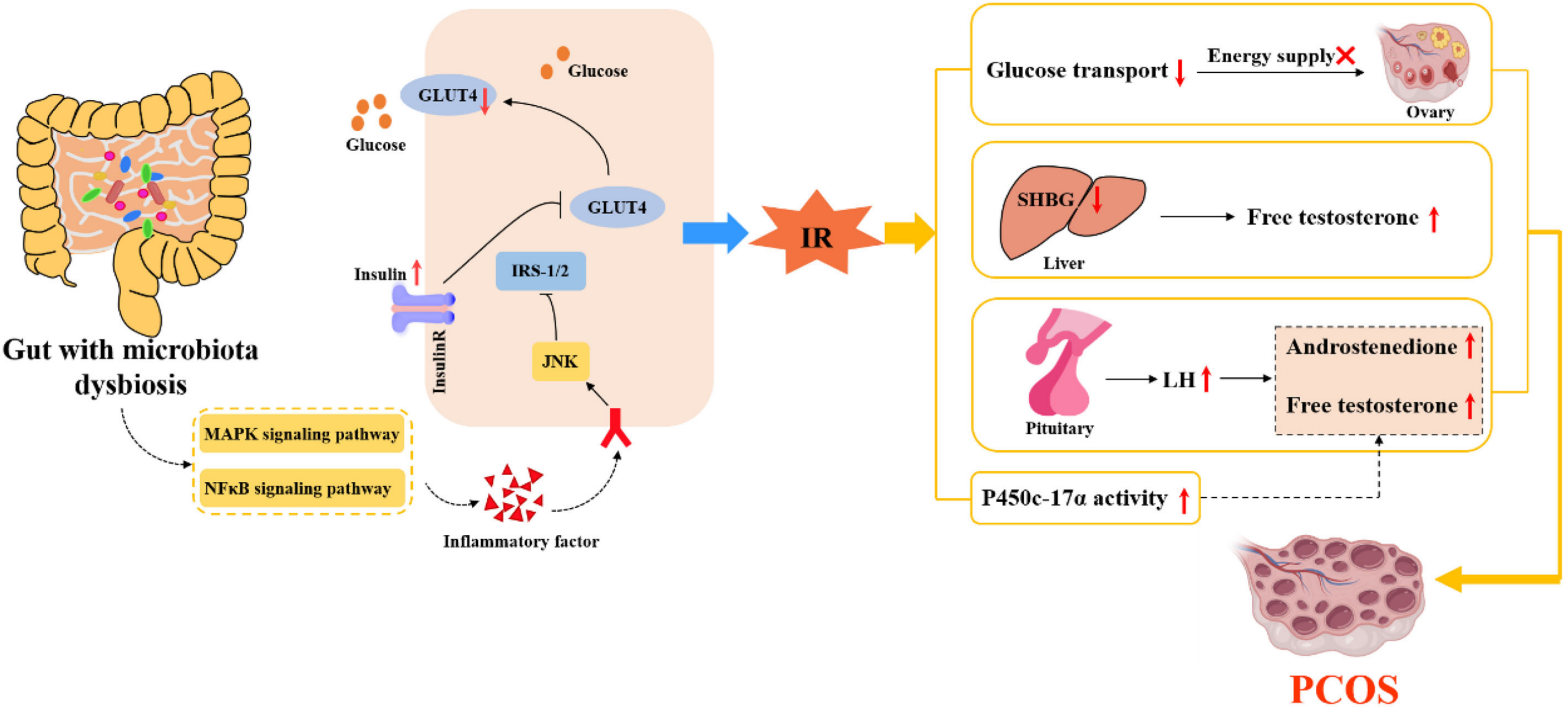
The internal relationship between IR, intestinal flora and PCOS.

In 2004, Gordon research team in the United States transplanted intestinal flora of conventional mouse into germ-free mouse. In the same feeding conditions, the body fat of sterile mice increased and IR appeared, which was the first evidence that intestinal flora was related to IR (43). The study showed (44) that compared with normal adult women, PCOS patients had intestinal flora disorder, intestinal mucosal barrier damage, increased intestinal wall permeability, and significantly increased endotoxemia related indicators. Insulin sensitivity is increased in patients with metabolic syndrome who are transplanted with healthy human flora (45). One study showed that the degree of tyrosine phosphorylation of insulin receptors in PCOS patients with IR was significantly lower than that in PCOS patients without IR, which indicated that PCOS patients with IR had defects in their own insulin receptor phosphorylation (46). It has also been suggested that intestinal flora can also affect insulin sensitivity through the inflammatory response mediated by branched amino acids (BCAAs) (15). Some scholars (47) revealed the relationship between intestinal flora and BCAAs, and found that *Prevotella* in human gut was involved in the synthesis of BCAAs. Zhang CM et al. (48) found that the levels of leucine and valine in follicular fluid of PCOS patients with IR were significantly increased. In this regard, they speculated that the disorder of amino acid metabolism would aggravate IR by altering glucose metabolism or inducing inflammation. All the above studies suggest that IR is correlated with the disruption of intestinal flora. Changes in intestinal flora can lead to increased permeability of the intestinal wall and the production of endotoxin factors, which enter the systemic circulation to activate the immune system. Furthermore, the c-jun amino-terminal kinase signaling pathway can be activated by nuclear factor κB and mitogen-activated protein kinases signaling pathways. And then, it causes the increase of serine phosphorylation of insulin receptor substrate and the decrease of tyrosine phosphorylation, leading to the disorder of insulin metabolism and triggering IR (49). IR caused by intestinal flora disturbance leads to abnormal glucose metabolism, HA and follicular dysplasia of PCOS. IR, in turn, exacerbates the disruption of the intestinal flora, which eventually causes the ovaries to produce more androgens, affecting the normal development of follicles, as shown in **Figure 2**.

## 4 Relationship between intestinal microecological disorders and chronic inflammation in PCOS

### 4.1 Chronic inflammation and PCOS

The view that the patients with PCOS have chronic low-grade inflammation was first put forward by Kelly et al. (50), who believed that it was related to IR and abdominal obesity. In recent years, studies have shown that the chronic inflammatory state of PCOS is not only manifested in the infiltration of macrophages and lymphocytes in the local pathology of ovary of patients with PCOS, but also the changes in the level of relevant inflammatory factors *in vivo*, such as hypersensitive C-reactive protein, interleukin-6, tumor necrosis factor, etc (50, 51). Inflammatory factors can affect insulin signal transduction by activating JNK, changing the phosphorylation level of insulin receptors, and blocking the expression of glucose transporter 4 (52, 53). In addition, the activation of JNK also promotes NF-κB signaling pathway in the islet, which in turn stimulates the production of more pro-inflammatory cytokines. Therefore, this vicious cycle of inflammatory cytokines will lead to islet β-cell dysfunction (54). In addition to inducing IR through a common signaling pathway, inflammatory factors can also stimulate the body to produce a large number of reactive oxygen species, which directly damage the insulin beta-cell sensitive to ROS, resulting in the reduction of the number of insulin beta cells or loss of function, and then lead to the occurrence of IR (55). Some studies have confirmed that the degree of oxidative stress and the level of inflammatory factors in patients with PCOS are positively correlated with the level of androgen (56). More directly, various inflammatory factors can trigger the production of excessive ovarian androgens or inhibit the aromatization of androgen into estrogen (57, 58). Obesity is a metabolic state characterized by chronic inflammation. In obese women with PCOS, the levels of some inflammatory mediators such as TNF-α, IL-6 and CRP are high, and they aggravate the inflammatory state of patients with PCOS by activating IKK signaling pathway (59, 60). In conclusion, the increased expression levels of some pro-inflammatory cytokines in patients with PCOS are also closely related to metabolic disorders such as IR, HA and obesity. Therefore, the pro-inflammatory cytokines have a certain influence on patients with PCOS directly or indirectly, and interact with other factors to aggravate the disease of PCOS.

### 4.2 Relationship between intestinal flora and chronic inflammation in PCOS

**Figure 3:**
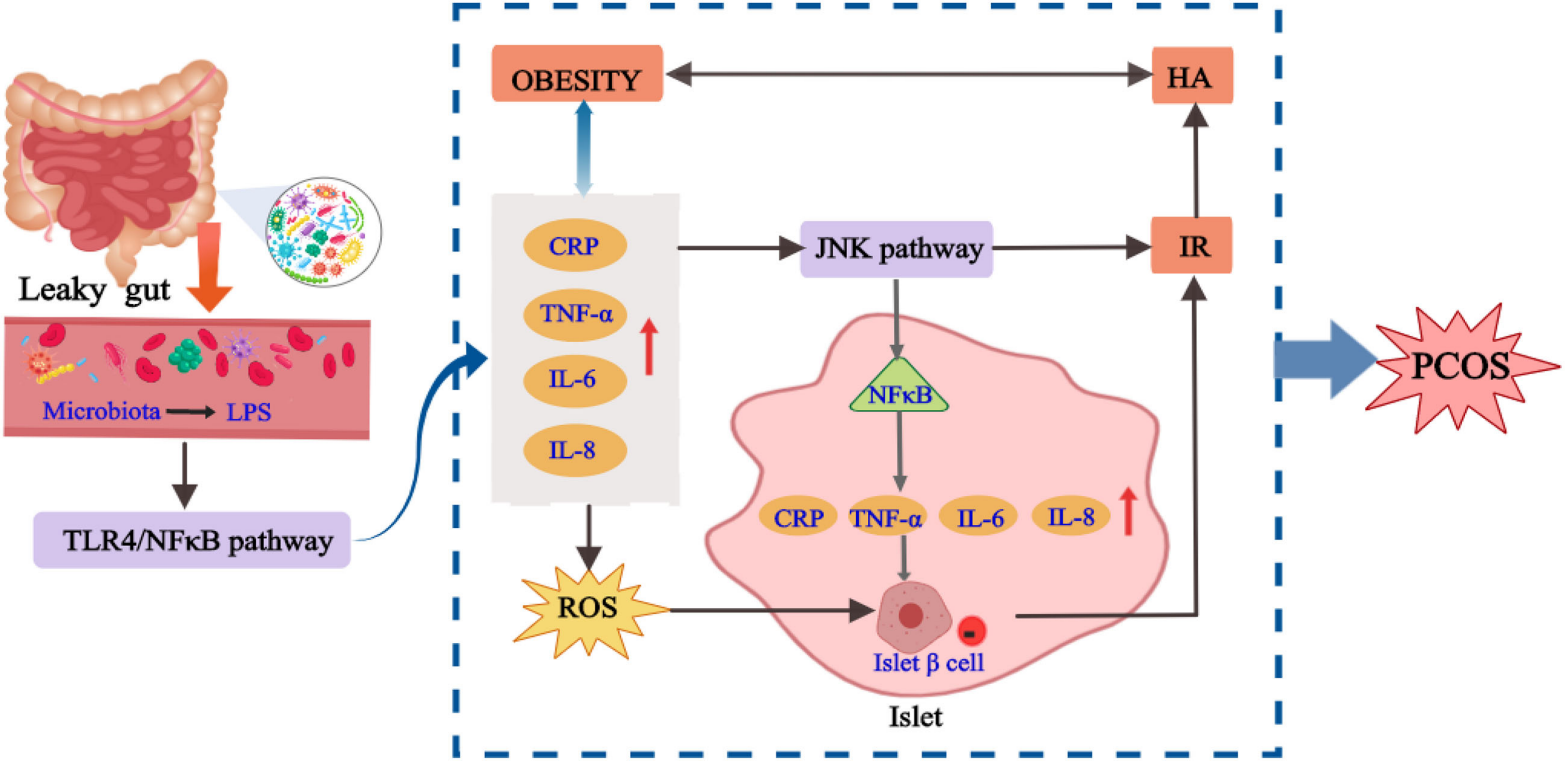
The internal relationship between chronic inflammation, intestinal flora and PCOS.

The occurrence of chronic inflammation is related to the changes of intestinal flora to some extent. The inflammatory factors generated by chronic inflammation may directly act on the hypothalamic-pituitary-gonadal axis, thus affecting the process of follicular development, maturation and ovulation in patients with PCOS. Cani et al. (61) proposed for the first time that “endotoxemia” produced by intestinal flora may be an important factor to initiate inflammatory activities. Xue et al. (62) found that in the PCOS mouse model induced by DHEA and high fat, the ovarian inflammatory indexes including TNF-α, IL-6 and IL-17A in the inulin group were significantly reduced compared with the model group. According to the sequencing and analysis of intestinal flora, compared with the model group, the number of *bifidobacterium* in the inulin group was increased. The correlation analysis also proved that intestinal flora was related to inflammatory factors, and inulin can alleviate the inflammatory state of PCOS by anti-inflammatory and improving intestinal microflora. A high fat diet for 4 weeks increased plasma LPS concentrations by two to three times, and this critical point is called metabolic endotoxemia. Endotoxemia caused by intestinal flora imbalance may be an important factor in the development of inflammation-mediated obesity and IR (61). When the ecosystem of intestinal flora is unbalanced, the intestinal permeability will increase, which enables LPS to enter the systemic circulation through the damaged intestinal mucosal barrier (63). Then, the carrier protein transports LPS to the membrane and binds with CD14. It stimulates the expression and production of various inflammatory factors by activating TLR4, resulting in IR, which then participates in the HA and metabolic abnormalities of PCOS (64). In conclusion, the inflammation state mediated by intestinal flora plays a significant role in the pathological process of IR and PCOS. There is a cross action between inflammatory signaling pathway and insulin signaling pathway. Endotoxemia caused by intestinal flora imbalance may be the key cause of inflammation, IR, HA and obesity (65), and the possible mechanism is shown in **Figure 3**.

## 5 Relationship between intestinal microecological disorders and obesity in PCOS

### 5.1 Obesity and PCOS

Obesity is a disease with excessive body fat, which is closely related to IR and increases the risk of metabolic diseases, including type 2 diabetes and cardiovascular disease (39, 66, 67). Obesity is not unique to PCOS, but women with PCOS are at a higher risk of overweight, obesity, central obesity and weight gain compared to non-PCOS women (68). Compared with overall obesity, obesity in PCOS is mostly abdominal obesity, that is, fat mainly accumulates in the abdominal wall and omentum. In the case of obesity and other metabolic disorders, the production and secretion of various fat factors (such as leptin) are significantly changed, and their functions are also substantially impaired. Obesity usually exacerbates PCOS through the production of adipokines and the reduction of SHBG (66). On the one hand, adipose tissue secretes adipokines that directly induce IR and adrenal androgen overload, leading to HA and hyperinsulinemia, or abnormal release of gonadotropin (elevated LH/FSH ratio) from the hypothalamus, leading to ovarian dysfunction, which in turn leads to PCOS-related HA and ovulation dysfunction. On the other hand, obesity can inhibit the synthesis of SHBG in the liver, thus promoting the secretion of androgen and insulin, which leads to IR, while high levels of insulin and androgen further aggravate the abnormal fat distribution. At the same time, obesity aggravates the metabolic dysfunction of PCOS patients, which leads to more prone to IR (69–71). Hence, obesity and PCOS are mutually causal. Obesity in adolescence can cause irregular menstruation and thin ovulation, thus promoting the occurrence of PCOS, while obesity in PCOS patients can lead to more serious HA, IR and other endocrine disorders (72).

### 5.2 Relationship between intestinal flora and obesity in PCOS

**Figure 4:**
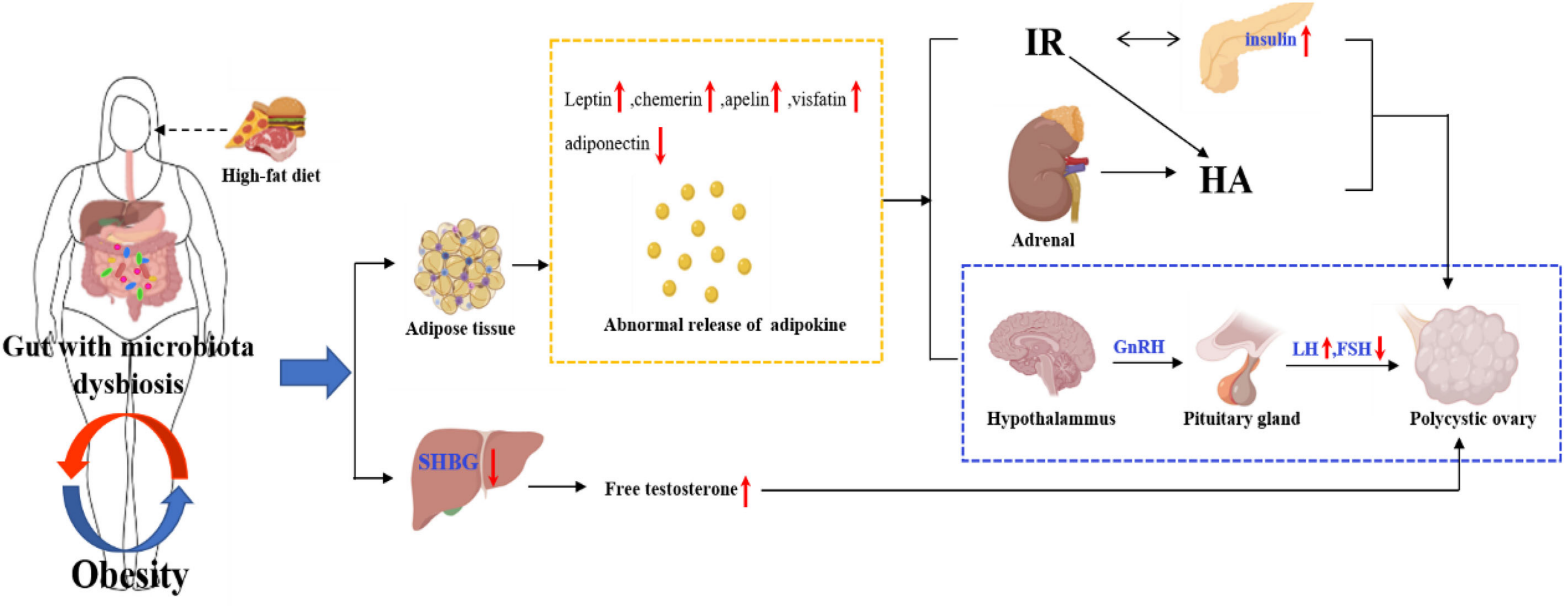
The internal relationship between obesity, intestinal flora and PCOS.

The imbalance of intestinal flora will directly affect the metabolism and immunity of the host and induce metabolic diseases of the host. Intestinal flora plays an important role in the development of obesity (73). By observing the changes in the weight of mice fed with sterile high-fat diet and normal low-fat diet, it was found that the weight increase of mice fed with high-fat diet was significantly lower than that of normal mice, which indicated that intestinal flora played a key role in the process of diet-induced obesity (74). A study found that leptin can reduce the weight of mice fed with high-fat diet, and its mechanism was mainly related to the increase of the diversity of intestinal flora and the reduction of endotoxin content, thus reducing the inflammatory state (74). The occurrence of obesity is mainly related to the high-fat diet. The intestinal flora is disturbed under the high-fat diet, resulting in the increase of LPS production and the decrease of SCFAs production (75). Modern research has found that the imbalance of intestinal flora may lead to obesity and lipid metabolism disorder through the SCFAs and G protein coupling, bile acid metabolism (76) and LPS pathway (65). The content of SCFAs in obese patients with PCOS is lower than that in healthy women. SCFAs coupled with GPR41 and GPR43 can promote the secretion of PYY and GLP-1 by intestinal L cells, which can delay gastric emptying and increase satiety, thus controlling the intake of diet and improving the abnormality of glucose and lipid metabolism (77). Therefore, the disturbance of intestinal flora will lead to the decrease of GLP-1 secretion, accelerate gastric emptying through the stimulation of gut-brain axis, improve the appetite for food, and then affect glucose and lipid metabolism, causing IR and obesity. Meanwhile, the disturbance of intestinal flora in PCOS patients increases LPS with endotoxin function and changes intestinal permeability. The increased LPS in the blood causes endotoxemia and chronic inflammation of the body, affects the function of insulin receptor, and then leads to IR and obesity (78). In addition, some intestinal flora, such as *Firmicutes* and *Bacteroidetes* (79), can encode bile brine hydrolyzase, which can metabolize primary bile acid into secondary bile acid. The composition of bile acid pool is changed by the farnesoid X receptor and the G protein-coupled bile acid receptor 1, or by dehydrogenation, dehydroxylation and epimerization, thus affecting the lipid metabolism and glucose homeostasis (80). The high-fat diet will lead to the imbalance of intestinal flora in patients, which will further affect the metabolism of glucose and lipid in patients and promote metabolic diseases such as obesity. Obesity can aggravate the IR and androgen level of PCOS patients, which may further exacerbate the metabolic disorder in PCOS patients, as shown in **Figure 4**. Therefore, weight loss through improving diet structure, exercise, surgery and other methods can effectively correct intestinal flora disorders (81). Improving intestinal permeability and alleviating IR can ameliorate the symptoms of HA and metabolic disorders in PCOS to a certain extent.

## 6 Discussion

The relationship between intestinal flora and PCOS has gradually become the focus of many studies in recent years. In 2012, Tremellen et al. (63) first proposed the hypothesis that PCOS is related to intestinal flora, suggesting that the imbalance of intestinal flora is associated with various manifestations of PCOS, such as HA, multiple ovarian cysts, and anovulation. Since then, the research on the relationship between intestinal flora and PCOS has started. Some studies have found that the overall diversity of intestinal flora is significantly different between patients with PCOS and healthy people, mainly reflected in the reduction of α diversity (82). Other studies have found that in the intestinal flora of patients with PCOS, specific microflora have changed, such as the change of the balance between *Bacteroides* and *Firmicutes* (83, 84), which will affect the production of short-chain fatty acids and have a negative impact on metabolism, intestinal barrier integrity and immunity. Liu et al. (85) conducted a controlled study of patients with PCOS and healthy subjects to explore the correlation between PCOS and intestinal flora. The 16SrRNA sequencing data showed that the intestinal flora diversity of patients with PCOS was lower than that of healthy people. The increase of relative abundance of *Firmicutes* and *Bacteroidetes* was positively correlated with androgen, body mass index and IR (86). In addition, after transplanting the intestinal flora of adult male mice into juvenile female mice, Markle et al. (37) found that the testosterone level in juvenile female mice increased. This indicated that the change of intestinal flora will affect the level of androgen in the serum of female mice, and indirectly participate in the occurrence and development of PCOS. Thus, intestinal flora may become a new therapeutic target for PCOS.

**Figure 5:**
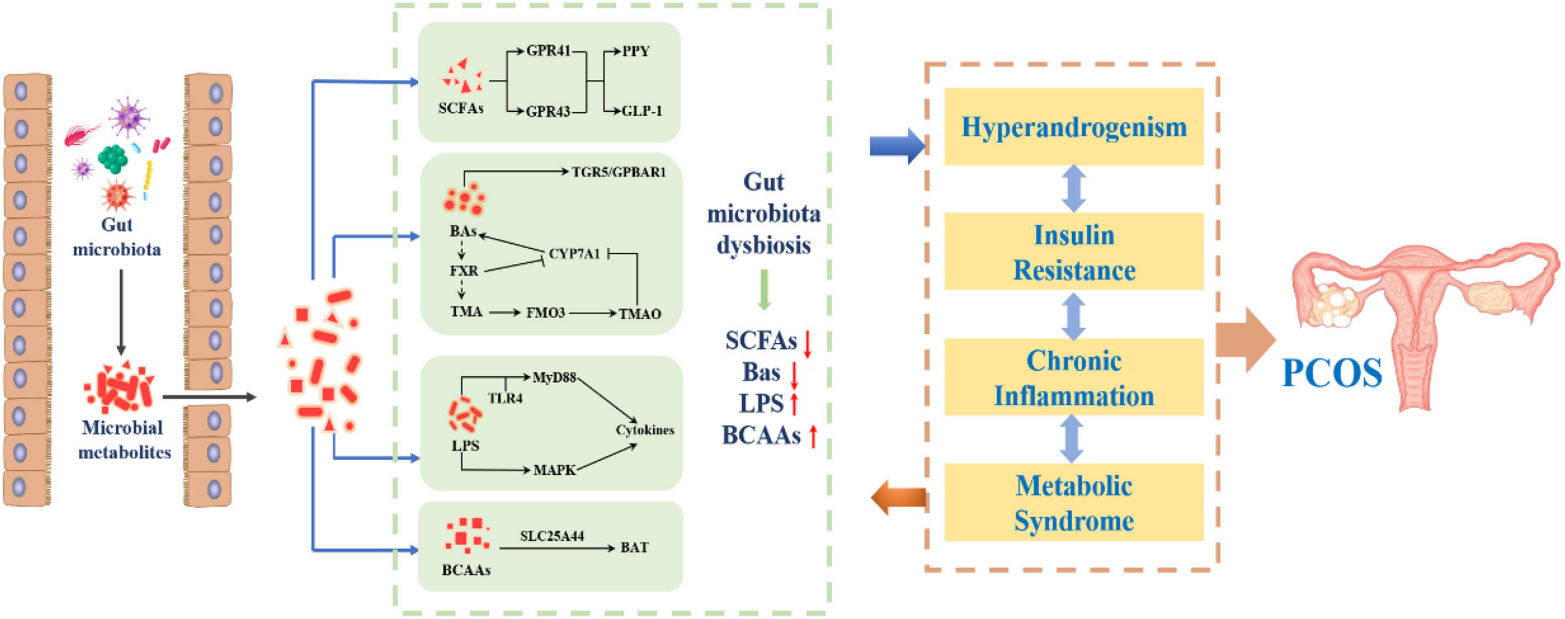
The internal relationship between intestinal flora and PCOS.

Although the relationship between the change of intestinal flora and PCOS has been found, there is no consensus on which bacteria are most relevant to PCOS, and the causal relationship between the two is not yet clear (87). Intestinal flora is the “endocrine organ” to maintain human health. The microbiota in the gut affects the reproductive endocrine system by interacting with estrogen, androgen, insulin, etc (63). A prospective study (88) involving 24 patients with PCOS and 19 healthy women confirmed that endotoxemia caused by gastrointestinal leakage was related to chronic inflammation, IR, fat accumulation and HA through 16S rRNA gene amplification and sequencing analysis. Kimural et al. (89) found that the body fat rate of GPR43-deficient mice was greatly increased, whereas the mice with increased GPR43 were still thin even after being fed a high-fat diet. This suggested that after ingestion of food, the body transmits signals through GPR43, a G-protein-coupled receptor for short-chain fatty acids (SCFAs), to produce and release energy to various tissues. As a protective barrier of intestinal microecology, SCFAs trigger the secretion of glucagon-like peptide through GPR43 and act on pancreatic islet P cells to regulate the production of insulin in the body, thus affecting the metabolism of PCOS. Bile acid, as a compound synthesized from cholesterol, can effectively help the utilization and digestion of lipids in the body’s lipid metabolism to improve the accumulation of lipids. However, bile acid in the gut, under the stimulation of SCFAs, can promote the synthesis and secretion of incretin by intestinal cells to participate in the regulation of blood glucose (90). Zhang J et al. (91) found that the patients with PCOS have a positive effect on the production of butyric acid by improving the homeostasis of intestinal flora and stimulating the secretion of lactic acid, which promotes the regulation of intestinal microorganisms and improves metabolic disorders. Intestinal flora can act on the gut brain axis through gastrointestinal hormones and other mediators, and regulate the central nervous system by mediating the release of hypothalamic gonadotrophin releasing hormone (GnRH), which can aggravate the progression of PCOS (92). Currently, more than 20 of the 50 gastrointestinal hormones are known to be involved in brain-gut axis interactions (93). In conclusion, intestinal flora disorders are involved in endotoxemia, the production of SCFAs, bile acid metabolism, brain-gut axis and other processes, which are related to HA, IR, chronic inflammatory response, obesity and other manifestations of PCOS (94). Therefore, intestinal flora may participate in the pathogenesis of PCOS by influencing follicular development, sex hormone and metabolic level through HA, IR, chronic inflammation and obesity, as shown in **Figure 5**.

## 7 Conclusion

As a complex endocrine and metabolic disorder, PCOS is closely related to HA, IR, chronic inflammatory, obesity, etc. The intestinal flora not only affects the metabolism of androgen, but also leads to IR, inflammatory reaction and obesity, which plays a key role in the occurrence and development of PCOS. In the past, genetic factors were considered to be one of the important causes of PCOS, but so far, no exact pathogenic genes have been found. As exogenous genetic material, intestinal flora will inevitably communicate with the host’s own genetic information, which will change the expression of host genes and trigger PCOS. It is precisely because the pathogenesis of PCOS is still unclear, so the treatment of PCOS is limited to the improvement of clinical symptoms such as IR, HA and ovulation disorders, rather than the radical treatment. However, with the deepening of research, the role of intestinal flora in PCOS will be gradually revealed, which is bound to provide new therapeutic strategies for PCOS.

## Author contributions

JL prepared the first draft of the manuscript. YL supervised the work and reviewed the manuscript. XL conceived the paper and revised the manuscript. All authors reviewed the manuscript and approved the submitted version.
